# A narrative review of the clinical applications of tiletamine-zolazepam in canine and feline anesthesia

**DOI:** 10.3389/fvets.2026.1807278

**Published:** 2026-06-16

**Authors:** Tzu-Yang Hung, Jin Yudong, Paulo V. Steagall, Laure Poincelot

**Affiliations:** 1 Shanghai Naughty Family Pet Co. Ltd, Shanghai Naughty Family Animal Hospital, Shanghai, China; 2 Dongdi Animal Hospital (Beijing) Co. Ltd, Beijing Dongdi Animal Hospital, Beijing, China; 3 Department of Veterinary Clinical Sciences, Jockey Club College of Veterinary Medicine and Life Sciences, City University of Hong Kong, Hong Kong SAR, China; 4 VIRBAC SA, GM&MD Department, Carros, France

**Keywords:** anesthesia, anesthetic, cat, dog, tiletamine, zolazepam, zoletil

## Abstract

Tiletamine-zolazepam is a widely utilized anesthetic combination in canine and feline medicine for chemical restraint, anesthetic induction, and minor surgical procedures. This narrative review synthesizes current evidence regarding the pharmacology, clinical applications, dosage regimens, and adverse effect profiles of tiletamine-zolazepam. Tiletamine functions as a non-competitive N-methyl-D-aspartate receptor antagonist, while zolazepam modulates gamma-aminobutyric acid neurotransmission. The combination provides a rapid onset of anesthesia, though recovery profiles vary significantly between species due to differing metabolic rates.

In cats, intramuscular tiletamine-zolazepam is administered for immobilization and minor surgery; however, its use is contraindicated in cats with cardiorespiratory, hepatic, or renal disease. Clinical dosages are generally lower than manufacturer label recommendations, particularly for intravenous administration, which requires titration to clinical effect. In dogs, tiletamine-zolazepam is indicated for short procedures or as an induction drug prior to inhalant maintenance. Hemodynamic studies demonstrate dose-dependent increases in sympathetic tone and potential respiratory depression, necessitating peri-anesthetic monitoring, airway protection, and the integration of multimodal analgesia.

Various combinations of tiletamine-zolazepam with ketamine, xylazine, dexmedetomidine, and opioids are commonly reported in veterinary medicine, offering predictable anesthesia but occasionally resulting in prolonged recovery. While tiletamine-zolazepam maintains a wide safety margin in healthy animals, it is contraindicated in those with head trauma, increased intracranial pressure, or pregnancy. This review underscores the necessity of balanced anesthesia and monitoring to optimize animal safety.

## Introduction

General anesthesia is characterized by analgesia (i.e., lack of pain perception), loss of consciousness, muscle relaxation and amnesia; there are usually not achieved by a single anesthetic alone. Balanced anesthesia protocols integrate analgesia, narcosis and muscle relaxation. Therefore, drug combinations are used in anesthesia with different drugs acting at different receptors and parts of the central nervous system, often in a synergistic manner (1, 34, 35). Injectable anesthetic drugs can be administered intramuscularly (tiletamine-zolazepam, ketamine, alfaxalone) or intravenously (all mentioned for intramuscular injection and propofol) to induce and maintain anesthesia in cats and dogs.

This narrative review aims to provide an overview of studies involving tiletamine-zolazepam (TZ) including dosage regimens, adverse effects, contraindications, and its clinical application in canine and feline anesthesia. This review synthesizes current literature regarding the clinical applications and dosage protocols of tiletamine-zolazepam in canine and feline anesthesia. To enhance the clinical relevance of this review, documented evidence is integrated with the authors' clinical observations.

## Methods

The methodology for this review utilized a qualitative, iterative approach to synthesize evidence-based data regarding the clinical application of tiletamine-zolazepam in canine and feline anesthesia. Primary literature was identified through a search of the PubMed and Web of Science databases, encompassing the period from 1969 to 2026. The search strategy used Boolean operators to compile core drugs and descriptors, using combinations of the following terms: “tiletamine AND zolazepam,” “Zoletil,”, “dog,” “canine,” “cat,” “feline,” and “anesthesia.” Selection criteria prioritized peer-reviewed original research, clinical trials and retrospective studies documenting objective physiological parameters and induction and recovery characteristics. While primary emphasis was placed on high-quality evidence published within the last 20 years, historical manuscripts were included to provide a comprehensive overview of the drug profiles. To ensure clinical utility, anecdotal reports lacking precise dosages or clinical data were excluded. Studies focusing on non-domestic species were generally omitted unless they provided essential pharmacological context. A consultation of supplementary sources, including specialized veterinary textbooks, doctoral theses, and conference proceedings helped solidify the search strategy. Additionally, a “snowball” sampling method was employed to identify further relevant literature from the reference lists of selected articles. The authors' collective expertise was integrated to bridge the gap between theoretical data and practical application. This approach aims to provide clinical guidance to support evidence-informed decision-making.

### Mechanism of action and pharmacology

Tiletamine is a dissociative anesthetic drug and likely has a similar mechanism of action to ketamine, a non-competitive antagonist of the N-methyl-D-aspartate (NMDA) receptors (2). The drug disrupts the binding of glutamate, a key excitatory neurotransmitter, leading to a functional dissociation of the cortex from sensory input, depression of the thalamocortical, limbic and reticular activating systems. There is evidence that the NMDA antagonism is associated with the prevention and treatment of central and peripheral sensitization (3). However, dissociative anesthetic drugs should never be used as stand-alone drugs for clinical pain relief (4). They can induce discomfort, muscle rigidity (i.e., catalepsy), loss of sensation and tremors when administered alone (5, 32).

Zolazepam is a benzodiazepine agonist that potentiates the effects of the inhibitory neurotransmitter gamma-amino-butyric acid (GABA) at the GABA receptor (6). Benzodiazepine agonists like zolazepam bind to specific GABA receptor subtypes, allosterically modulating GABAergic neurotransmission and consequently decreasing neuronal excitability, responsible for centrally-mediated muscle relaxation and sedation (7). The commercial formulation of tiletamine-zolazepam is presented as a powder for reconstitution with 50 mg/mL of tiletamine and 50 mg/mL of zolazepam. The dose is determined by the total combined concentration (100 mg/mL).

Tiletamine-zolazepam is rapidly absorbed following intramuscular (IM) or intravenous (IV) administration. The high degree of lipophilicity exhibited by both drugs facilitates their rapid distribution across the blood-brain barrier and into the central nervous system. Tiletamine has a high volume of distribution, suggesting extensive tissue penetration beyond the vascular compartment (8). Zolazepam is rapidly distributed after administration, including into adipose tissue, which can serve as a reservoir for prolonged drug effects (2). However, there is a lack of original pharmacological studies using tiletamine-zolazepam. Textbooks describe that both drugs undergo hepatic biotransformation and are primarily excreted by the kidneys (2, 9). Therefore, it should be expected that the duration of anesthesia and recovery time may be prolonged in animals with hepatic and renal disease (10, 11). The pharmacology of tiletamine-zolazepam is different between cats and dogs; zolazepam has a longer half-life than tiletamine in cats and the opposite is observed in dogs (12). This may explain some prolonged but better anesthetic recoveries after tiletamine-zolazepam in cats than in dogs (13). The onset of anesthesia is rapid, typically occurring within 2 to 5 min after IM administration. The duration of anesthesia is expected to range between 30 and 60 min, which is variable depending on the specific dosage regimen and the use of concurrent drug combinations.

### Clinical recommendations, contraindications and animal selection in both species

Tiletamine-zolazepam is indicated for chemical restraint (immobilization), short-duration minor surgical procedures or sedation pre-euthanasia, requiring mild-to-moderate analgesia, and anesthetic induction (14, 32). When administered as a monotherapy, tiletamine-zolazepam can induce dose-dependent adverse effects, including muscle rigidity, hypersalivation, and tremors (12). Higher dosages are positively correlated with an increased frequency and severity of these complications (15, 16). To refine the safety profile and clinical efficacy of tiletamine-zolazepam, the adoption of balanced anesthetic protocols is strongly recommended. The integration of opioid agonists alongside alpha-2 adrenoceptor agonists and local anesthetic blocks facilitates robust multimodal analgesia and superior muscle relaxation. These combinations exert a “sparing effect,” allowing for a significant reduction in the required drug volume while mitigating inherent adverse effects (17) (18, 19).

In veterinary practice, analgesia should be proactively addressed with opioids and non-steroidal anti-inflammatory drugs (NSAIDs), tailored to the intensity and duration of the surgical stimulus (14). Regardless of the administration route, anesthetic requirements are significantly influenced by the drugs and dosage regimens selected for premedication, and the type, intensity, and duration of the painful stimulus (14). For IV administration, clinical experience suggests lower dosages, typically 1 to 2 mg/kg, delivered slowly over 1 min and titrated to the desired clinical effect. Conversely, IM administration generally requires higher dosages, ranging from 3 to 6 mg/kg. The onset of anesthesia is rapid (2 to 5 min), with a clinical duration of 30 to 60 min, highly contingent upon the specific dosage regimen and concurrent drug combinations used. Regardless of the administration route, perioperative monitoring, airway protection, and preoxygenation are recommended. In non-intubated patients, supplemental oxygen administration should be considered to maintain stable oxygen level and mitigate the risk of hypoxemia (20). Tiletamine-zolazepam should be used with extreme caution in patients with pre-existing cardiorespiratory disease, such as hypertrophic cardiomyopathy, hyperthyroidism, or pulmonary edema. (17, 21). Furthermore, tiletamine-zolazepam is contraindicated in patients with head trauma or increased intracranial pressure, as dissociative drugs can elevate cerebral blood flow and perfusion pressure (Maddison et al., 2008). In cases of hepatic or renal insufficiency, impaired drug metabolism and excretion may result in significantly prolonged recovery phases (12).

There remains a notable lack of prospective, randomized studies regarding tiletamine-zolazepam use in pediatric and geriatric populations. Therefore, it is recommended to adjust the dosage of tiletamine-zolazepam based on the individual condition of the animal. Tiletamine-zolazepam crosses the placental barrier, which may result in respiratory depression in neonates. To the authors knowledge, there are currently no prospective, randomized study using tiletamine-zolazepam for cesarean section in dogs or cats. Consequently, until further high-level evidence becomes available, its use for these specific obstetric procedures is not recommended.

### Clinical application in domestic cats

According to the label, tiletamine-zolazepam is indicated for chemical restraint or general anesthesia combined with muscle relaxation (intramuscular administration only) in cats. Tiletamine-zolazepam is popular in feline practice for procedural sedation (immobilization) and surgery, particularly involving minor procedures (14). The combination of tiletamine-zolazepam with opioids, local anesthetics and alpha-2 adrenoceptor agonists aim to mitigate adverse effects through better analgesia, sedation and muscle relaxation, and allowing the use of lower doses of tiletamine-zolazepam.

Tiletamine-zolazepam combinations should be reserved to healthy cats and avoided in cats with cardiorespiratory disease that may not tolerate respiratory depression and increases in sympathetic tone (e.g., hypertrophic cardiomyopathy, hyperthyroidism, pulmonary edema).

Early studies investigating the hemodynamic effects of intravenous and high doses of tiletamine-zolazepam (9.7, 15.8, and 23.7 mg/kg) showed dose-dependent effects on blood pressure and cardiac output (15). Significant increases in blood pressure, peripheral vascular resistance, and cardiac contractility were observed post-administration, persisting between 10 and 90 min; notably, heart rate did not change significantly during this period. Respiratory acidosis was observed due to respiratory depression as reflected in arterial pH and blood gas results without changes in arterial oxygen tension. Cats became apneic after the two highest doses justifying why tiletamine-zolazepam should never be administered at any of these doses intravenously (15).

Tiletamine-zolazepam can produce muscle rigidity, hypersalivation, respiratory depression and prolonged anesthetic recovery (12, 22). Based on the authors' clinical experience, atropine should not be used to reduce hypersalivation as it can further increase blood pressure, heart rate and myocardial oxygen consumption. Poor muscle relaxation was reported using tiletamine-zolazepam alone (15 mg/kg IM) for feline ovariectomy (22, 23) (Table 1).

**Table 1:** Summary of anesthetic protocols involving tiletamine-zolazepam combinations in domestic cats.

Anesthetic protocol	Drug mixture	Route of administration	Comments
TZ + K + X (TZKX)	TZ powder is reconstituted with 100 mg of X (100 mg/mL) and 400 mg of K (100 mg/mL) Dose of 3.3 mg/kg (normally between 0.25–0.27 mL/cat)	IM	Anesthetic-induced mortality of feral cats without known comorbidities was 0.23% Reversal with 0.5 mg/cat of yohimbine Analgesia is recommended with opioids, local anesthetics and NSAID
TZ + K + X (modified TZKX)	250 mg TZ reconstituted with 300 mg of K and 50 mg of X; each mL 38.4 mg of TZ, 46.2 mg of K and 7.7 mg of X Mean volumes of administration of 0.45–0.47 mL/cat than the classic TZKX (0.25–0.27 mL/cat)	IM	Anesthetic-induced mortality of feral cats without comorbidities was 0.3% Reversal with 0.5 mg/cat of yohimbine Analgesia is recommended with opioids, local anesthetics and NSAID
TZ + methadone	TZ (3 mg/kg) in combination with methadone (0.2 mg/kg) No additional mix than reconstitution of TZ	IM	Heart and respiratory rates, time to extubation and sternal recumbency were similar between groups, but time to standing was longer with TZ - methadone than acepromazine - methadone.
TZ + D + butorphanol or nalbuphine	TZ is reconstituted to a concentration of 100 mg/mL using 2.5 mL D (0.5 mg/mL) and 2.5 mL of either nalbuphine or butorphanol (10 mg/mL) (usually 0.03 mL/kg of TZD + opioid) TZD with opioids IM (3 mg/kg TZ, 7.5 μg/kg D and either butorphanol or nalbuphine at 0.15 mg/kg)	IM	Analgesia is recommended with additional doses of opioids, local anesthetics and NSAID An error in the published protocol by Kreisler et al. (19) has been corrected with the author's agreement. The corrected reconstitution is as follows: TZ is prepared to a final concentration of 100 mg/mL by adding 2.5 mL of dexmedetomidine (0.5 mg/mL) and 2.5 mL of either nalbuphine or butorphanol (10 mg/mL).

IM, intramuscular; D, dexmedetomidine; K, ketamine; TZ, tiletamine-zolazepam; X, xylazine; NSAID, non-steroidal anti-inflammatory drug.

The use of other routes of administration (subcutaneous and oral transmucosal) may induce significant variability in the physiological and anesthetic effects and increased likelihood of undesirable effects (36). For example, the oral transmucosal administration of tiletamine-zolazepam (5–7.5 mg/kg) resulted in hypersalivation in the majority of cats receiving the 7.5 mg/kg dose. Furthermore, the onset of anesthesia was up to 15 min and dysphoric behavior was observed during anesthetic recovery (36). The subcutaneous route is considered unreliable, frequently leading to excitatory effects without producing effective anesthesia (22).

In the context of neutering, tiletamine-zolazepam has been reported in kittens, with a recommended IM dose of 11 mg/kg for 6- to 14-week-old male kittens (37). However, poor muscle relaxation was observed and drug combinations should be considered. In feline blood donors, anesthesia was typically achieved using a tiletamine-zolazepam at 5 mg/kg. There was minimal impact on standard blood variables and blood was collected at 10 mL/kg (up to a maximum volume of 60 mL) (38).

### Drug combinations in domestic cats

Tiletamine-zolazepam in combination with ketamine and xylazine (TZKX) has been used for general anesthesia of feral cats before neutering. The TZKX has become popular in shelter medicine. The anesthetic effects are usually predictable with rapid onset of action (e.g. 1–2 min) when a small volume of administration is given and with the benefit of partial reversibility using yohimbine. A large retrospective study reported no anesthetic complications following TZKX use. However, anesthetic monitoring was limited to assessment of mucous membrane color, respiratory and heart rate (39). In a separate prospective study involving approximately 100 cats receiving TZKX combination, desaturation (defined as SpO2 ≤ 90% at least once) was observed in most cats. Supplemental oxygenation is recommended when using this specific combination. The TZKX was not associated with hypotension, bradycardia, or mortality in a prospective study; mild hypothermia was observed in female cats (36.6 ± 0.8 °C in female at the time of reversal compared to 38.0 ± 0.8 °C in males). On the other hand, prolonged recoveries were reported, even following intravenous yohimbine administration (median time of 72 ± 42 min from reversal to sternal recumbency) (40). Shorter recovery times (20 to 40 min) after yohimbine administration has been reported when a modified TZKX combination is administered (41) (Table 1).

However, re-administration of TZKX during surgery prolonged recovery times (exceeding 100 min). In one previous study, approximately 16.8% of cats (969 out of 4,584 cats) required a second dose during surgery. It is important to contextualize this finding, as the prolonged surgical duration in that study may have been a result of the involvement of veterinary students within the spay-neuter program (39). The modified TZKX, which was associated with shorter recovery, required additional doses in up to 23.9% of female cats (152 out of 636 cats). Tiletamine-zolazepam combinations have evolved over time to include better analgesia with the addition of opioids. For example, lower doses of tiletamine-zolazepam (3 mg/kg) in combination with methadone (0.2 mg/kg) IM was administered before neutering. This drug combination produced better chemical restraint and sedation for venous catheterization when compared with acepromazine (0.03 mg/kg) – methadone (0.2 mg/kg).

Tiletamine-zolazepam has been combined with dexmedetomidine (TZD) and either butorphanol or nalbuphine (17–19). The TZD + opioid has an onset of action of approximately 5–10 min and recovery times of approximately 10–20 min. However, cats often required maintenance anesthesia with isoflurane during the surgery (19).

### Clinical application in dogs

Tiletamine-zolazepam is indicated for chemical restraint and minor surgical procedures of short duration (up to 30 min) that require mild to moderate analgesia via IM route. The IV use of tiletamine-zolazepam in dogs is specifically indicated for the induction of anesthesia followed by maintenance with an inhalant anesthetic.

For anesthetic induction of non-premedicated dogs, a dose of approximately 3.5–4 mg/kg is typically required intravenously for successful endotracheal intubation (25). Based on the authors' clinical experience, the IV dose is often lower and administered to effect (typically 1–2 mg/kg) when tiletamine-zolazepam is part of a multimodal protocol with other sedatives and opioids. Conversely, higher doses (up to 5 mg/kg) are commonly administered via the IM route. In this case, sternal or lateral recumbency is typically observed within 5 to 10 min following administration (26, 27).

An evaluation of the hemodynamic and respiratory profiles of tiletamine-zolazepam at 6.6, 13.2, and 19.8 mg/kg demonstrated rapid anesthetic induction (11–16 s) and dose-dependent recovery times (16). Induction time (mean ± standard deviation) was 16.0 ± 4.8, 15.3 ± 3.8, and 11.2 ± 3.2 s for the 6.6, 13.2, and 19.8 mg/kg doses, respectively. Tiletamine-zolazepam administration induced an increase in heart rate across all doses, whereas marked elevations in cardiac output were restricted to the two highest dosage groups. Systemic vascular resistance decreased following administration of the two highest doses. Clinically significant respiratory depression occurred exclusively at the 19.8 mg/kg dose, characterized by a reduction in min ventilation from a baseline of 8.9 ± 3.4 L/min to 4.2 ± 1.3 L/min at 1-min post-injection; this reduced min ventilation persisted for the 90-min duration (2.7–3.9 L/min). Conversely, lower dosages resulted in only transient or negligible ventilatory deviations (16).

In a canine model of acute hemorrhagic hypovolemia (30 mL/kg blood volume depletion) anesthetized with low-concentration sevoflurane, a 10 mg/kg IV bolus of tiletamine-zolazepam induced transient apnea. Notably, despite this respiratory effect, cardiovascular stability was maintained, with heart rate, mean arterial pressure (MAP), and cardiac output remaining within baseline limits (28). These results suggest that tiletamine-zolazepam may be considered as an alternative protocol for hypovolemic dogs when general anesthesia is maintained with low concentrations of an inhalant anesthetic. The use of tiletamine-zolazepam could be particularly advantageous when low doses (2–3 mg/kg) are used to mitigate the risk of respiratory depression.

An investigation into the profiles of IM tiletamine-zolazepam (3 mg/kg) as a preanesthetic drug prior to propofol induction (3 mg/kg) demonstrated robust sedative efficacy, with 67% (4/6) of dogs achieving sternal recumbency within 4–6 min post-administration (26). This protocol facilitated rapid endotracheal intubation and maintained relatively stable hemodynamics. While mean carotid arterial pressure increased following the administration of tiletamine-zolazepam and TZ-propofol combination, the observed biphasic heart rate response did not differ significantly between TZ-propofol and propofol monotherapy (6.5 mg/kg). Although the combination extended the time to regain sternal recumbency (29.2 ± 4.3 min vs. 22.3 ± 3.4 min for propofol alone), the respiratory impact remained within clinically acceptable parameters. Despite a 29.8% increase in end-tidal concentration of carbon dioxide with TZ-propofol, contrasting a 6.5% increase with tiletamine-zolazepam alone, the values were maintained within a physiological range of 38–44 mmHg. During the recovery phase, dogs exhibited characteristic hypersalivation and transient myoclonus or muscular hypertonicity, typically persisting for 10–15 min after return to sternal recumbency. These findings suggest that the combination offers effective chemical restraint and a significant propofol-sparing effect with manageable physiological deviations.

While premedication with tiletamine-zolazepam may be utilized prior to anesthetic induction with propofol, the incorporation of adjunctive drugs is indicated to have an adequate muscle relaxation throughout the procedure, and facilitate a rapid anesthetic recovery.

In comparison with other anesthetic drugs, hemodynamic changes similar to those described for tiletamine-zolazepam monotherapy were observed in sevoflurane-anesthetized dogs, and overall, cardiovascular and metabolic effects were similar between tiletamine-zolazepam, alfaxalone and ketamine-diazepam (25). Recovery quality was similar between tiletamine-zolazepam and ketamine-diazepam but inferior to propofol and alfaxalone (29).

Regarding the impact on intraocular pressure, in one study, intravenous tiletamine-zolazepam at doses of 5, 10, and 20 mg/kg did not produce significant changes in canine intraocular pressure (30, 33). These findings were corroborated in a separate study comparing IV tiletamine-zolazepam (5 mg/kg) with propofol (8 mg/kg). In both groups, intraocular pressure remained within the usual range, but it was significantly higher with propofol than with tiletamine-zolazepam following anesthetic induction and endotracheal intubation (31). Considering these results, tiletamine-zolazepam may be considered an option for anesthetic induction in dogs undergoing ocular procedures, particularly when there are pre-existing concerns regarding increased intraocular pressure.

### Drug combinations in dogs

Tiletamine-zolazepam has been studied in combination with other drugs in dogs (Table 2). As described in the cat section, the tiletamine-zolazepam, butorphanol and dexmedetomidine (TZBD) combination has been used in the context of spay-neuter programs in dogs (18, 20, 21). Favorable clinical properties included rapid onset of anesthesia (3–5 min to lateral recumbency), rapid immobilization, predictable effects, low volume of injectable (0.01 to 0.03 mL/kg) and the concept of “one-size-fits-all” approach using the intramuscular route of administration (18). The hemodynamic effects of an intramuscular injection of TZBD combination did not differ significantly regardless of whether ketamine was included in the protocol. This combination (TZBD) induced hypoventilation and respiratory depression, characterized by a rise in the partial pressure of carbon dioxide increasing to 58–75 mmHg and a corresponding decline in arterial pH (7.20–7.25). Both protocols (TZBD with or without ketamine) resulted in hypertension (MAP frequently exceeding 160 mmHg) and increased systemic vascular resistance accompanied by reflex bradycardia (often lower than 50 bpm). Despite these changes, cardiac output, oxygen delivery and oxygen consumption remained comparable to values observed during sevoflurane anesthesia, and blood L-lactate concentrations remained within the reference range (20, 21).

**Table 2:** Summary of anesthetic protocols involving tiletamine-zolazepam combinations in dogs.

Anesthetic protocol	Drug mixture	Route of administration	Comments
TZ + B + D	TZBD admixture TZ = 100 mg/mL B = 10 mg/mL D = 0.25 mg/mL 0.005–0.015 mL/kg for mild to moderate sedation 0.02 to 0.025 mL/kg for mild to moderate painful procedures 0.03 mL/kg for surgery	IM	Short onset of anesthesia (2–3 min), rapid immobilization, predictable effects, low volume of injectable (0.01 to 0.03 mL/kg) Concept of “one-size-fits-all” approach using the intramuscular route of administration. Analgesia supplemented with additional doses of opioids, local anesthetics and NSAIDs
TZ + K + X	TZKX admixture TZ = 100 mg/mL K = 80 mg/mL X = 20 mg/mL 0.025 mL/kg	IM	Addition of methadone to provide analgesia (Methadone: 0.3 mg/kg)

IM, intramuscular; IV, intravenous; B, butorphanol; D, dexmedetomidine; K, ketamine; TZ, tiletamine-zolazepam; X, xylazine; NSAID, non-steroidal anti-inflammatory drug.

The TZBD admixture was prepared by reconstituting 500 mg of tiletamine-zolazepam lyophilized powder with a 5.0 mL diluent mixture. This diluent mixture consisted of 2.5 mL of butorphanol (10 mg/mL) and 2.5 mL of dexmedetomidine (0.5 mg/mL). The resulting solution achieved final concentrations of 100 mg/mL tiletamine-zolazepam, 5 mg/mL butorphanol, and 0.25 mg/mL of dexmedetomidine.

The tiletamine-zolazepam, ketamine and xylazine (TZXK) admixture was prepared by reconstituting 500 mg of tiletamine-zolazepam lyophilized powder with a 5.0 mL diluent mixture. This diluent consisted of 4.0 mL of ketamine (100 mg/mL) and 1.0 mL of xylazine (100 mg/mL). The resulting solution achieved final concentrations of 100 mg/mL tiletamine-zolazepam, 80 mg/mL ketamine, and 20 mg/mL xylazine. The administration of the TZKX combination at a dose of 0.025 mL/kg resulted in a dosage regimen of tiletamine–zolazepam (2.5 mg/kg), ketamine (2 mg/kg) and xylazine (0.5 mg/kg) (24). The addition of methadone (0.3 mg/kg) to the TZKX protocol provides supplemental analgesia without compromising anesthetic depth. Heart rate and respiratory rate decreased from baseline in both groups (HR: TZKX: 123 ± 21 to 93 ± 23 bpm; TZKX + Methadone: 106 ± 20 to 80 ± 18 bpm; RR: TZKX: 40 (30–72) to 24 (16–29) bpm; TZKX + Methadone: 42 (24–56) to 16 (11–30) bpm). Median sedation/anesthesia scores were identical between groups, and median time to lateral recumbency was 5 (4–6) min (TZKX) vs. 6 (4–10) min (TZKX + Methadone) (24).

### Future perspectives in both species

While tiletamine-zolazepam remains a common drug of choice for canine and feline anesthesia, future research should prioritize prospective, randomized clinical trials specifically targeting high-risk populations, such as patients classified as ASA III and IV. There is a clear imperative to optimize ultra-low dose tiletamine-zolazepam protocols through the integration of novel sedative and analgesic adjuncts. Furthermore, the current existing literature is predominantly focused on healthy patients undergoing routine gonadectomy. Data from studies have been derived from studies using experimental healthy dogs, and the impact of comorbidities on body homeostasis, particularly in patients undergoing concurrent isoflurane or sevoflurane anesthesia, requires further investigation. Consequently, investigations are required in geriatric and pediatric patients, as well as those with diverse coexisting pathologies or undergoing complex surgical procedures. Refining evidence-based dosing strategies for these vulnerable populations would help to mitigate recovery complications and ensure cardiovascular stability. Ultimately, bridging the gap between historical empirical success and standardized, contemporary protocols remains the primary objective for advancing the safety and efficacy of procedural sedation in canine and feline medicine.

## Conclusion

This review summarizes the clinical indications, pharmacological profiles, and contraindications of tiletamine-zolazepam in canine and feline anesthesia. While tiletamine-zolazepam is effective for immobilization, induction, and minor procedures, its use as a monotherapy is often associated with increased muscle rigidity and dose-dependent respiratory depression and heart rate increase. To mitigate these effects and ensure potent analgesia, the integration of opioids, local anesthetics, and alpha-2 adrenoceptor agonists within a balanced anesthetic framework is essential. Although specific contraindications exist, most notably in animals with cardiorespiratory, intracranial, or severe metabolic disease, tiletamine-zolazepam remains a common drug of choice and versatile tool in veterinary medicine. When combined with modern multimodal strategies and vigilant monitoring, tiletamine-zolazepam-based protocols offer rapid, predictable, and effective anesthesia for feline and canine patients.

## Data Availability

The raw data supporting the conclusions of this article will be made available by the authors, without undue reservation.
